# P.A.D.D.L.E.: a hypothesis generation tool for assessing pollution’s potential role in disease

**DOI:** 10.1038/s41598-026-39836-2

**Published:** 2026-02-13

**Authors:** Grace Ratley, Aditi Vijendra, Jalin Jordan, Pranav Thota, Jordan Zeldin, Prem Prashant Chaudhary, Ian A. Myles

**Affiliations:** 1 Epithelial Therapeutics Unit, National Institute of Allergy and Infectious Disease, National Institutes of Health, Bethesda, MD USA; 2 Unit of Integrative Metabolomics, Institute of Environmental Medicine, Karolinska Institute, Stockholm, Sweden

**Keywords:** Diseases, Environmental sciences, Risk factors

## Abstract

Since the 1960s, tens of thousands of chemicals have been added to the global market, yet the vast majority lack comprehensive health risk assessments. During this same period, industrialized nations have experienced dramatic increases in inflammatory diseases, raising concerns about environmental contributors. We aim to provide a tool for researchers to explore associations between environmental toxicant releases and diseases of interest, assess impacts of the route of exposure, connect findings to protein targets and biological pathways, map geographic “hot spots”, and identify at-risk populations. We employ multivariate, nonspatial elastic net regression and univariate, spatial mixed effects modeling to assess connections between 61.9 million health care visits for 5,984 diagnoses and 6-year averaged exposures to 571 air and 42 water pollutants across 16,451 zip codes. Economic confounders including regional deprivation indices and healthcare access metrics were included. Demographic disparities in environmental exposure burdens were also assessed. Our analysis revealed that diseases of neurodevelopment and epithelial inflammation were most frequently linked to mechanistically plausible toxic exposures. Ultimately, we created an interactive web tool, Pollution Associated Disease Diagnosis Likelihood Estimator (P.A.D.D.L.E.), that provides epidemiological associations to guide future research. We emphasize that these associations are based on observational data and require further validation studies to establish causal disease-toxicant relationships.

**Supplementary Information:**

The online version contains supplementary material available at 10.1038/s41598-026-39836-2.

## Introduction

The rapid, modern rise in inflammatory diseases among industrialized nations has prompted an increased concern for the role of environmental toxicants in generating the rise in chronic diseases. These include allergies^1^, autoimmunity^2^, cancer^3,4^, metabolic disorders^5^, neurodevelopmental disorders^6^, and more. Since the 1960s, over 340,000 new chemicals have been identified^7^; tens of thousands of which have unknown impacts on human health but are nonetheless common components of the global market^8^. However, mapping associations between chemical exposures and diseases can present significant challenges. For example, toxicants are often co-linear, such as with the traffic related air pollutants (TRAPs)^9^. Thus, a disease with a causal link to nitrogen dioxide may have statistical links to other, non-causal, TRAPs. Yet, simultaneously assessing disease risks against multiple toxicants is computationally and time intensive, especially given that the route of exposure may influence toxicity. Given that many cell or animal models often require multi-condition studies to establish dose and time responses, diseases with numerous associations may prove logistically challenging without a means to prioritize the associations most worthy of investigation. Similarly, the best tools for assessing biomarkers of exposure (such as hair, blood, or urine concentrations) are still only capable of detecting a limited range of chemicals within a single experiment^10^. Even when a primary candidate toxicant for a given disease is identified, the mechanistic pathways through which the chemical may impact pathology may not be well established.

While modeling based on individual-level exposure data would provide higher confidence associations between toxicants and disease risk than can be achieved with aggregated data, such approaches face significant limitations. The collection of individual-level data presents prohibitive logistic and financial barriers, and the necessarily limited sample sizes would preclude comprehensive assessment of the thousands of diagnoses available within large-scale registry data. We previously demonstrated the translational value of an aggregated multi-toxicant exposure approach for atopic dermatitis (AD) and psoriasis, identifying specific toxicant associations which facilitated targeted mechanistic validations^11–17^. In brief, the prior work started with a hypothesis of AD being driven by chemical exposures, then: used spatial and non-spatial modeling to identify chemical associations with skin disease; evaluated pathways impacted by those chemicals which overlap with AD pathogenesis to prioritize the most informative links; tested these chemicals in models using human cells, mice, and skin commensal bacteria to establish mechanisms; tested the influence of real-world products containing these chemicals; and then applied post-hoc analysis to clinical trial data based on local exposure levels^11,12,14,18,19^.

While this work successfully started with a disease of interest to identify toxic associations, it was incapable of starting with a chemical of interest to assess disease associations. Therefore, a tool which can simultaneously assess a variety of diseases against numerous toxicants, gauge the impact of the route of exposure, and suggest mechanistic plausibility through linking correlated toxicants to established protein targets and identifying enriched biological pathways would greatly benefit the research community. Furthermore, differences in chemical exposures across ethnic and income groups is well established in the US^20^; incorporating the impact of historical redlining and regional poverty may elucidate environmental injustice.

Herein, we present the first tool to facilitate simultaneous, untargeted assessments for the associations between numerous diagnoses and chemical exposures. In total, our analysis performed both spatial and nonspatial penalized regression modeling to assess connections between 61.9 million health care visits for 5,984 total diagnoses and 6-year averaged exposures of 571 air and 42 water pollutants in 16,451 zip codes. Although this tool looks at thousands of diagnoses, it was designed to aid biomedical researchers focused on specific diseases of interest. P.A.D.D.L.E. offers the research community a tool for hypothesis generation and dealing with toxicant co-linearity. Ultimately, use of this tool may inform validation studies and human biomarker evaluations to help researchers wade through the increasingly complex nature of toxicant exposure data.

## Methods

### Derivation of disease rates

Disease rates were taken from the Agency for Healthcare Research and Quality (AHRQ) Synthetic Healthcare Database for Research (SyH-DR), accessible from https://www.ahrq.gov/data/innovations/syh-dr.html. The database collected all billable clinical visits in the United States which occurred in 2016. Rates for each International Classification of Diseases (ICD) included in the SyH-DR were calculated by taking the incidence of diagnosis and dividing by the total billed diagnoses for each included zip code. For each visit, it is possible that up to 10 ICD diagnoses were given in addition to a primary diagnosis. There are roughly 70,000 ICD-10 diagnostic codes which offer a high level of specificity regarding location, cause, and severity of a disease or injury as well as comorbidity. Consequently, for any given visit to a health care visit, several codes may have equal clinical validity as a primary diagnosis, with their ordering sometimes reflecting administrative rather than clinical priorities. We therefore counted each diagnosis separately instead of relying on primary diagnosis. This approach offers several advantages: it makes the models more resistant to arbitrary coding decisions, captures disease prevalence more completely, and creates an internal validation mechanism whereby toxicants correlated with multiple ICD codes for the same disease provide stronger evidence for association. However, we acknowledge this approach introduces a bias that inflates the apparent frequency of common, chronic, or multi-system diseases, such as diabetes, which generate multiple related diagnostic codes per visit. Multiple visits to a provider by the same patient were treated as separate, as this may suggest greater severity of symptoms.

The SyH-DR anonymizes diagnoses in zip codes with less than 10,000 people and the locations for disease diagnoses occurring less than 10,000 times. Therefore, these rare disorders and small towns were pre-excluded from the analysis. We further excluded ICD codes that occurred in less than 2% of zip codes to reduce the risk that an ICD would be used only by a few providers in a localized region. Converting ICD codes to their respective names was performed using the ICD code list provided by the Centers for Medicare and Medicaid Services (CMS) found at https://www.cms.gov/medicare/coordination-benefits-recovery/overview/icd-code-lists.

Unlike the prior models we used employing a smaller, private data set^11,12^, SyH-DR does not contain specific subspecialties of providers; thus, overall access to billing providers could be entered as a covariant but not access to specific subspecialist. Similarly, while depersonalized disease visit data could inflate rates for disorders that require frequent follow up, such data may capture severity and should not differ between zip codes. For example, the need for frequent follow up for a given ICD should be disorder intrinsic, and thus if a given zip code had significantly greater visit frequency for a specific ICD than others, such information would be worthy of causal inquiries.

### Derivation of pollution exposures

Air pollution exposure was derived from the EPA databases Risk-Screening Environmental Indicators (RSEI) and Toxics Release Inventory (TRI). O3, CO, SO2, NO2, PM10 and PM2.5 outdoor air concentrations were derived from the Center for Air, Climate, & Energy Solutions data base (CACES) using their Land Use Regression (LUR) model, and the census tract-level data was averaged for overlapping zip codes^21^. The exposures were collated from the years 2010–2016 to contrast with the AHRQ data from 2016. Water pollution was separately evaluated using the Monitoring Unregulated Contaminants in Drinking Water (UCMR) data from the EPA. The UCMR data from UCMR 3–5 was combined to contrast against the 2016 AHRQ data as well. If differing measurements of the same chemical were reported in different UCMR databases, the results were averaged prior to analysis. Feature matrixes were constructed by the same method as previously described^11,12,19^. In brief, for each of the zip codes in the AHQR database, we define a 30-mile catchment area around the zip code centroid. For all facilities within the catchment area, the total amount of each pollutant is summed. If there are no facilities releasing a particular pollutant in the catchment area, that zip code is assigned zero for that pollutant. Since the zip code represents the location of the outpatient provider, rather than the residence of the patient, a Gaussian distribution is defined to weight the relative importance of facilities based on distance from the zip code center, represented by the equation: ((N/ ∑ ι = 1) Pollutant_i_ * Population_i_ * N (dist_i_; 0, σ))/((N/ ∑ ι = 1) * Population_i_ * N (dist_i_; 0, σ)). The center of the distribution is the centroid of the zip code where the provider is located, and the standard deviation is 10.7785 miles, which is derived from the assumption that the mean distance between a patient and their provider is 8.6 miles^22^. Water pollution was counted only towards the zip code of the measurement device reported by the EPA. Although water pollution may cross zip codes via rivers and tributaries this data is not captured in the UCMR, thus limiting the analysis to zip codes that had monitors precludes assuming values for areas without known dissemination patterns.

### Derivation of confounders

For both spatial and non-spatial models, Deprivation Index was used as an economic cofounder as reported in the Neighborhood Altas from the Center of Health Disparities Research at the University of Wisconsin (https://www.neighborhoodatlas.medicine.wisc.edu/) were included in the model. Population density and age distributions (21 five-year age bins, as proportions) were included per the US Census of 2020 (https://www.census.gov/). In the non-spatial model, geographic coordinates (i.e. latitude and longitude) were also included.

### Model specification

We used two complementary modeling approaches to assess associations between environmental toxicant exposures and disease diagnosis rates: a non-spatial penalized regression and a spatial penalized regression. Code for both models is found in the Supplemental File. Both approaches were applied separately to each of 5,984 disease diagnoses. Nonspatial analysis was performed separately for air and water pollutants and across five age strata (pre-K: 0–5 years of age; pediatric: 6–17; adult: 18–54; retirement age: 55–74; and geriatric: 75+). Spatial modeling was only performed on air pollution and, due to the increased computational power required, across just two collapsed age strata (pediatric: 0–17 [combining pre-K and pediatric] and adult: 18+ [combining adult, retirement, and geriatric]).

For each disease and age group, the outcome variable represented the age-standardized diagnosis rate in a zip code to account for varying healthcare utilization and differentiating age-specific disease burdens. The predictor matrix for air pollution models consisted of 592 variables including: 571 environmental exposures (TRI, RSEI, and CACES) and 21 sociodemographic covariates (census age distribution [21 five-year age bins, as proportions], deprivation index, population density, and for the non-spatial model, latitude and longitude). The predictor matrix for the non-spatial water pollution model contained 42 environmental exposures and the same sociodemographic covariates. All predictors were z-scored prior to modeling to facilitate comparison of effect sizes across variables with different measurement scales.

Nonspatial analysis was performed as previously described^12,19^, using the glmnet package in R (R version 4.3.3 in R Studio version 2023.12.1 + 402)^23^. For each disease-age combination, we fit an elastic net regression model with an alpha of 0.5 and tuned the regularization parameter lambda for each model using 10-fold cross-validation, selecting the value which minimized mean squared error. Given the high dimensional predictor matrix, elastic net balances the goals of dimensionality reduction to identify meaningful toxicant-disease associations and accounts for multicollinearity of predictors while preserving their inclusion in the model (as opposed to an arbitrary selection as in Lasso).

For the purposes of visualization, we filtered associations that were less than two standard deviations (2SD) from the mean beta-values, rather than using a p-value filter as elastic net regression does not produce p-values. Strictly to aid in readability, correlations more than 5SD from the mean were displayed, but all correlations can be found within the website data.

For spatial modeling, we fit a negative binomial generalized linear mixed effects model with nested spatial random effects as previously described^11^. Unlike the elastic net, the spatial models use a univariate approach due to computational constraints of mixed-effects models. This allows for the evaluation of spatial autocorrelation (regional differences that may be unrelated to pollution) but sacrifices the ability to correct for correlations between toxicants. The outcome variable was the count of visits with the diagnosis in each zip code, with an offset for total visits in that age group added as a covariate. The model was implemented using adaptive Gauss-Hermite quadrature (nAGQ = 0, Laplace approximation) with the BOBYQA optimizer in the lme4 package. We created a four-level nested spatial hierarchy using hierarchical complete-linkage clustering on great-circle distances between zip code centroids, generating clusters of approximately 81, 27, 9, and 3 zip codes at each successive level. These nested clusters were included as random effects to capture spatial autocorrelation at multiple scales. Therefore, each model included one focal air pollutant, sociodemographic covariates (age distributions, population density, and deprivation index), 4 regional clusters as random effects, and an offset for total visits.

The use of zip code aggregated data for both clinical visits and exposure data introduces considerable biases including: the ecological fallacy, in which population level associations may not reflect individual risk; the zip code of the clinical visit may not be the same as the zip code in which the patient resides or works; and the arbitrary boundaries of zip codes as administrative districts may inflate results from urban centers with a high density of zip codes as well as introduce greater error into the exposures of zip codes with large geographical area. Despite these limitations, the use of aggregated data enables analysis of nationwide data while protecting individual privacy, provides sufficient spatial resolution to detect regional patterns, and aligns with how environmental exposure data are commonly aggregated and released. Our penalized regression approach and inclusion of spatial smoothing terms help mitigate some aggregation effects by borrowing strength from neighboring areas. Furthermore, this approach is consistent with the intended aim to design a hypothesis-generating tool to identify potential associations warranting further investigation with individual-level exposure assessment and longitudinal designs.

### Social determinant comparisons

Racial disparities were calculated by taking the percentage representation of each race/ethnicity from the US Census of 2020 (https://www.census.gov/). Historic redlining scores for 2020 US census tracts were used after registration from the Environmental Impact Data Collective (https://redivis.com/datasets/rnef-d56dafea8?v=1.0). Exposure rates for social determinants were collected from the years 2013–2019 to compare against the 2020 census.

### Product analysis

Connecting which commercial products contain any indicated chemical was taken from EPA ChemExpo databases (https://comptox.epa.gov/chemexpo/get_data/).

### Mapping toxicant exposures

Spatial and non-spatial modeling was performed as for diseases. Mapping functions were performed using the ggmap and viridis packages in R.

### Website design

The website was designed using R Shiny (version 1.10.0) and plotly (version 4.10.4).

### Pathway analysis/protein associations

Protein-toxicant interactions were accessed from the Toxin-Toxin-Target Database (T3DB; http://www.t3db.ca/)^24^. We performed protein-level enrichment analysis to identify proteins disproportionately targeted by disease-associated toxins. Using Fisher’s exact test, we assessed whether the number of disease-associated toxins interacting with each protein exceeded chance expectation. For example, if 10 of 21 disease-associated toxins interact with estrogen receptor 1, enrichment analysis determines whether this represents a statistically meaningful enrichment (suggesting a potential mechanistic link) or could occur randomly. Enriched proteins are indicated on the website if the FDR-corrected p-value of Fisher’s exact test is less than 0.05. Separately, the set of proteins with disease-associated toxicant interactions were used to perform a pathway enrichment analysis, which utilizes the enrichR package (version 3.4; Jawaid W, 2025) and references 4 databases: GO Biological Process 2018, GO Molecular Function 2018, KEGG 2019 Human, and WikiPathways 2019 Human. Like the protein enrichment above, enrichR uses Fisher’s exact test, which takes an unordered list of genes and computes pathway enrichment.

### Disease category derivation

The total number of diagnoses per ICD letter-code were tallied and converted to the percent of total diagnoses. For example, most ICD diagnoses were in the T-codes, for injuries. However, none of the coefficients for chemical exposures >2SD from the mean were within the T code (suggesting chemical exposure does not associate with accidents). By comparison, F codes (mental and behavioral health) represented only 5.6% of total diagnoses in patients under 18 years of age but represented 39.4% of the ICDs with coefficients >2SD from the mean.

### Data availability

All code and files required to run PADDLE, including all data presented in this manuscript, can be directly downloaded from GitHub: https://github.com/ratleyge/PADDLE. Spreadsheets indicating the levels of toxicant exposures by disease/zip code are accessible in this repository. Due to privacy protections, absolute visitation rates for each disease by zip code are not publicly available but can be obtained through direct application to the AHRQ. Relative visit rates expressed as percentiles by disease and zip code are available in the GitHub repository under PADDLE/Data/Disease_mapping_data. Those seeking access to the SyH-DR database to establish raw visit numbers per zip code must send their requests to AHRQ. References for all packages used in the analysis and website design can be found in the supplement.

### Databases

Land Use Regression // Air pollution // CACEST3DB // Toxicant and protein interactions // Univ of Alberta.

**Table:** 

Database Name	Information measured/accessed for model	Managing body
US Census	Population density, ethnic and racial demographics of zip code	US Census Bureau
ChemExpo	Home products known to contain toxicants	EPA
GO Processes	Genomic pathways	Open Biological Ontologies Foundry
KEGG	Genomic and metabolomic pathways	Kanehisa Laboratories
Neighborhood Atlas	Socio-economic disadvantage of region	Univ of Wisconsin
RSEI	Air and water pollution release by factories	EPA
SyH-DR	Visit rates for billed diagnostic codes by zip code	AHRQ
TRI	Air and water pollution release by factories	EPA
UCMR	Measured levels of water pollutants	EPA
Wikipathways	Genomic and metabolomic pathways	Wikipathway consortium

## Results

### In children, correlations between chronic diseases and pollution were enriched for neuro-developmental disorders

**Fig. 1:**
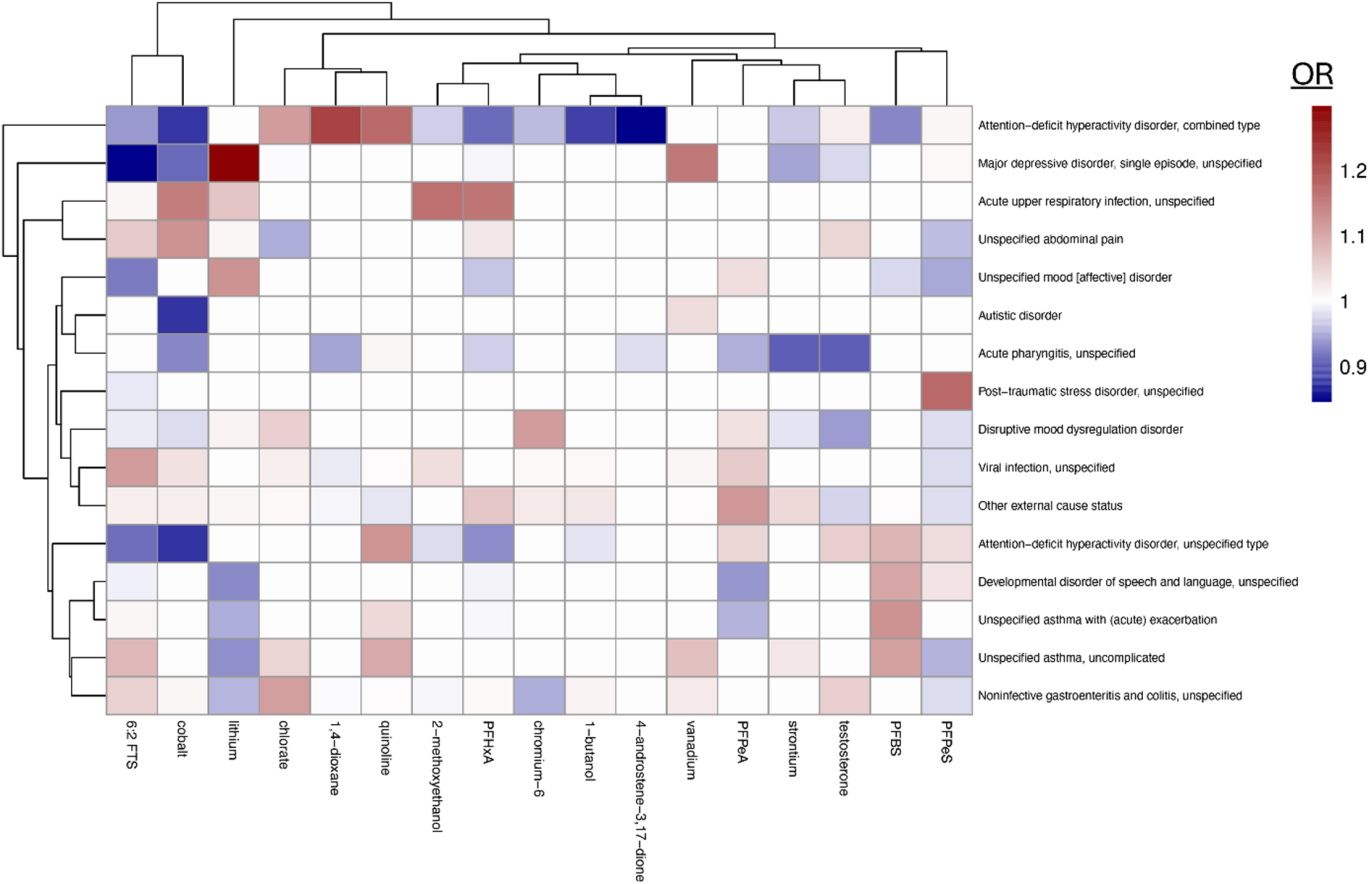
Associations between water pollution and diseases in children aged 6–17 years. Non-spatial correlations for water pollution and diagnosis rate for children aged 6–17 years by zip code displayed as odds ratio (OR). A mean value was derived for the OR between every disease and every variable across the entire cohort. Diseases containing OR that were greater than 5 standard deviations removed from the mean are displayed solely to aid in readability. All associations are included in the website.

Nonspatial modeling was performed for children of ages 0–5 years (pre-kindergarten; Pre-K) and those 6–17 years (Pediatric) for both air and water pollution exposures. Focusing on the associations which were more than five standard deviations from the mean, we identified several correlations. Many of the links were in neurodevelopmental, epithelial, or immunologic organ systems (Supplemental Table 1). Examples included Pre-K attention deficit disorder associated with phosphorous air pollution (Figure S1) or Pediatric links with 1,4-dioxane in the water (Fig. 1). For nonspatial analyses, evidence of reverse correlations was noted, such as links between latitude and various diseases (Figure S1). Such associations more likely reflect regional differences in access to care or diagnosis but were better accounted for using spatial analysis (Supplemental Table 2). A continued signal for epithelial and behavioral health disorders was identified under spatial assessments, however additional disorders of congenital development and hematologic disorders were implicated (Supplemental Table 2).

### Correlations with adult disease were consistent with established literature

Similar to the disorders identified as having strong pollutant associations in children, nonspatial analysis in adults identified behavioral and neurologic disorders (Supplemental Table 3; Figure S2-3 A). Addictions to controlled substances were also among the most strongly associated, which could indicate an impact on drug metabolism, neurologic function, or represent reverse causation from economic hardships generating both drug use and political tolerance for pollutant sources (Supplemental Table 4). Disorders with the largest coefficients (> 5SD from the mean) were most enriched for mental and behavioral disorders in both children and adults (Figure S3B). However, other significant associations were identified for disorders of female reproduction. For example, in accordance with reports from other nations^25,26^, NO_2_ was linked to breast, uterine, and pelvic cancer (Supplemental Table 4). Also consistent with prior reports^27,28^, the volatile pesticide 1,2,3-trichloropropane and the semi-volatile nitrosamine, N-nitrosodiophenylamine, were linked to abnormal fetal development. Multiple associations between chemical exposures and cancers were evident for all age groups (Figure S4A-B).

**Fig. 2:**
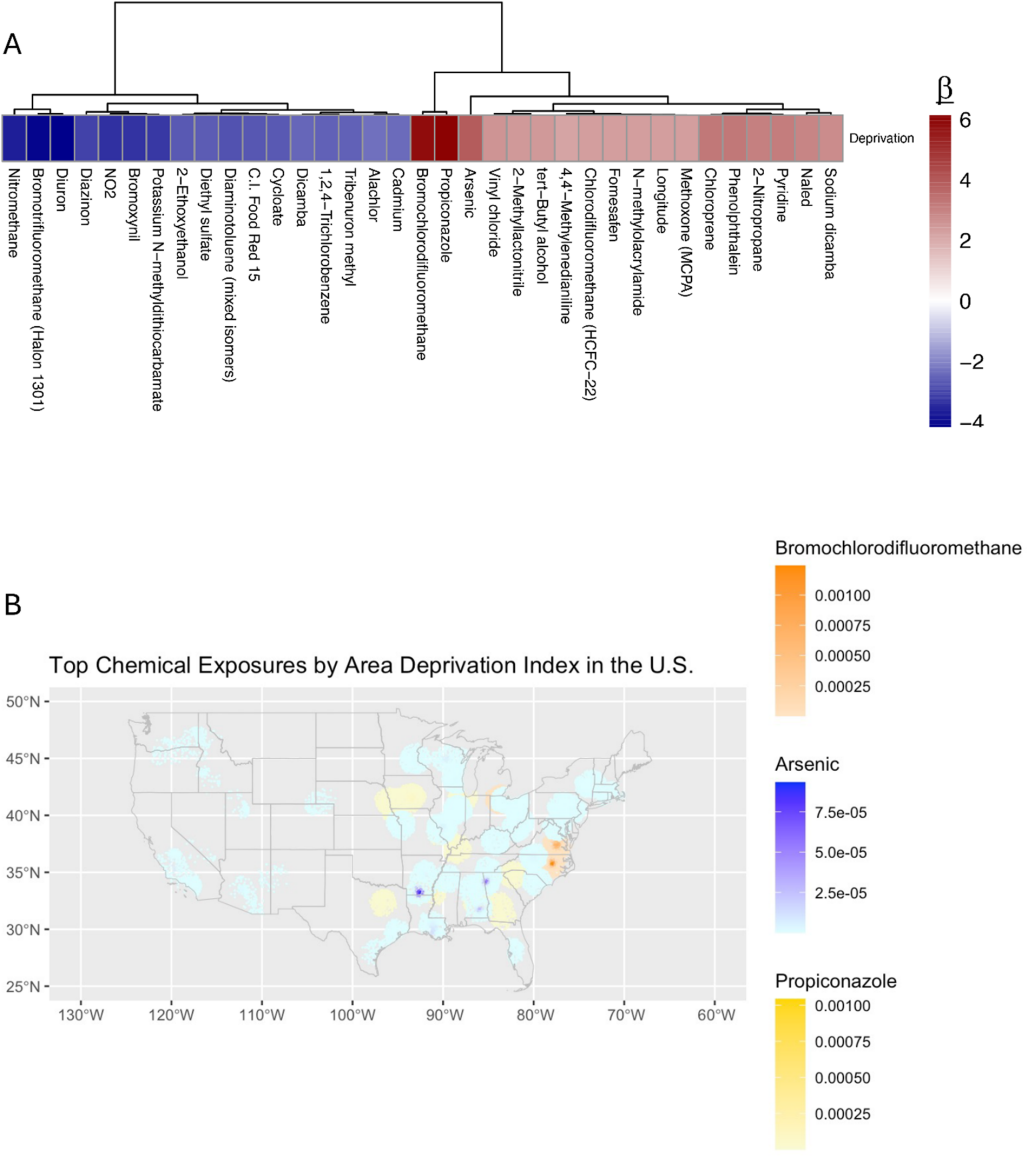
Associations between deprivation index and air and water pollution by zip code. Non-spatial correlations for air and water pollution versus the zip code deprivation index is displayed as odds ratio (OR). A mean value was derived for the OR between every disease and every variable across the entire cohort. (**A**) Diseases containing OR that were greater than 2 standard deviations removed from the mean are displayed. (**B**) Mapped concentrations for indicated toxicants which represent the three chemicals with the strongest associations with deprivation, indicated as mcg/m^3^ weighted by 30-mile catchment areas.

**Fig. 3:**
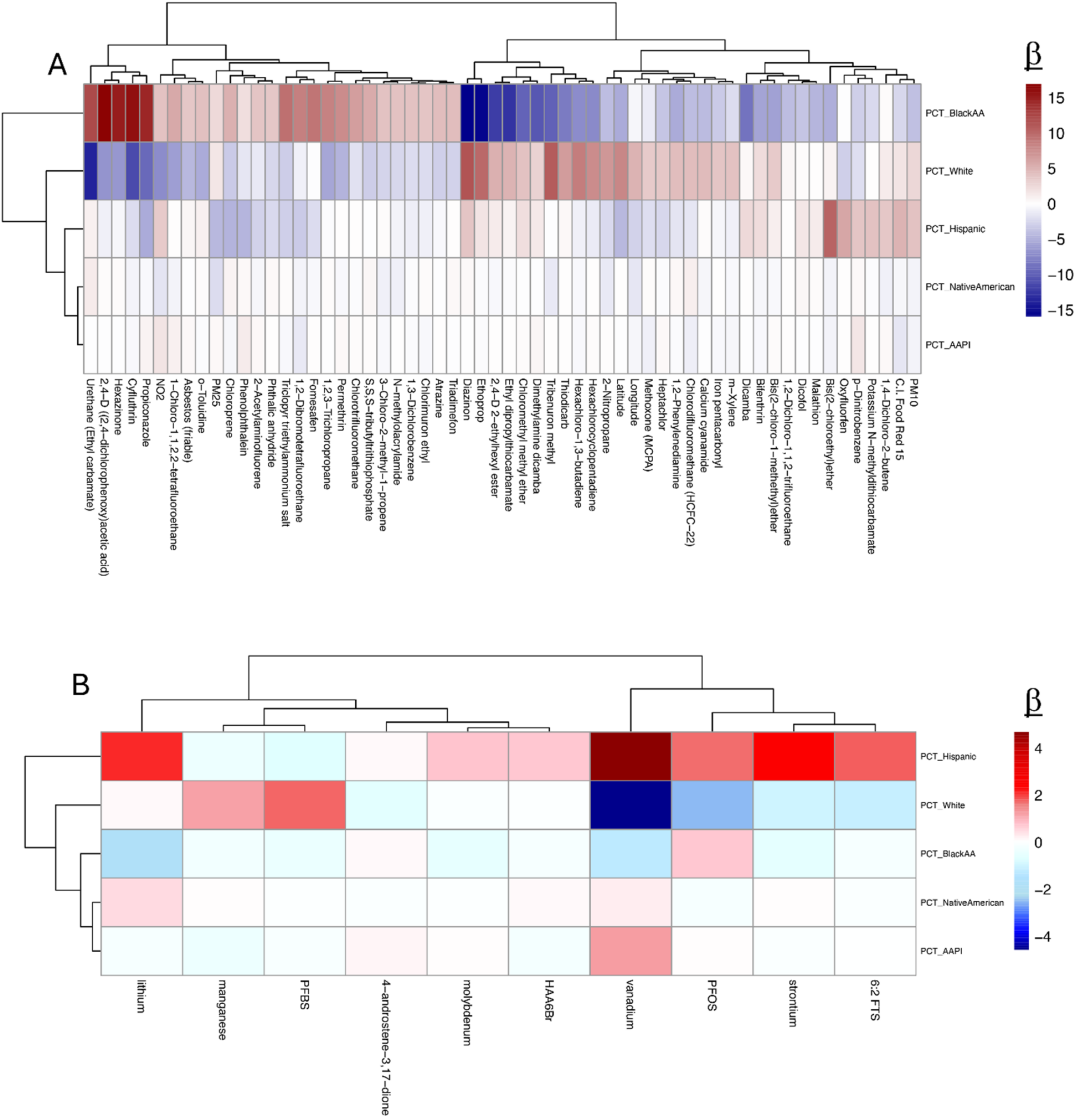
Associations between percentage of population of self-reported ethnicity and chemical exposures. (**A**) Non-spatial correlations displayed as beta coefficients (b) for chemical exposures from air pollution sources reported from 2013–2019 versus the percentage of the population with the self-described ethnicity in the 2020 census. (**B**) Non-spatial correlations displayed as beta coefficients (b) for chemical exposures from water pollution sources.

Sickle cell diagnoses were linked to NO_2_ and PM_2.5_, each of which have been previously linked to increased rates of emergency room visits for sickle cell crises^29^. Cystic fibrosis diagnoses were linked with crotonaldehyde, which has several reported pulmonary toxicities including indirect, in vitro effects on the cystic fibrosis transmembrane receptor via inhibition in epithelial sodium channels^30,31^. While pollutants would have no impact on the incidence of these monogenic disorders, these data may indicate risk for worsening control for such diseases, creating an increased need for healthcare interactions for these patient groups.

### Enumerating at-risk groups

Exposomics is “an integrated compilation of all physical, chemical, biological, and (psycho)social influences that impact biology”^32^. We thus assessed how financial, historical, and racial differences between zip codes were associated with chronic chemical exposures. The area deprivation index, a complex marker of regional poverty^33^, was associated with differential exposure to several chemicals (Fig. 2A). Deprivation indices correlated with increased exposures to propiconazole, bromochlorodifluoromethane, and arsenic but lower exposure to nitromethane, Halon 1301, and diuron (Fig. 2A). However, the exposure to these toxicants was unevenly distributed across the US (Fig. 2B).

Historical segregation and redlining limited America’s community integrations resulting in nonhomogeneous ethnic demographics of zip codes. To test our model’s ability to detect associations with disparities, we first identified an a priori set of variables. Our model correctly identified the regional distribution of ethnicities (i.e. Black American populations being a larger percentage of zip codes in the southeast and similarly for Hispanic Americans in the southwest). The approach also correctly identified known disparities^34^in exposure to NO_2_, PM_2.5_, PM_10_, and deprivation (Figure S5A). Associations between a zip codes racial composition and the regional chemical exposures identified that hierarchical clustering separates along the lines of Black Americans versus non-Black for air pollution including propiconazole, cyfluthrin, and hexazinone, 2,4-D, urethane, and HBCD (Figure S5B; Fig. 3A). For water pollution, the divide was between Hispanic and non-Hispanic populations with particular risk for heavy metals like vanadium, strontium, and lithium (Fig. 3B). Under a nonspatial analysis, chemical exposures for zip codes with increasing percentage of Black Americans appear inversely correlated with the exposures in zip codes with predominantly White populations (Fig. 3A). The reduced number of associations seen with spatial adjustments was led by the air quality index (AQI) related toxicants like CO, NO_2_, and PM_2.5_ (Figure S5B). However, living in mostly White areas did not protect against every exposure, and in fact conferred greater risk for chemicals including thiodicarb, diazinon, ethoprop, and PFBS (Fig. 3A-B).

Zip codes can be assigned a historical redlining score (HRS; a marker of whether the area was previously redlined, even if the region had subsequently undergone developmental change or redistricting). Exposure to potassium bromate was strongly linked with HRS but was geographically variable (Figure S6A-B). Racial disparities in the exposures to the chemicals which were a priori linked to allergic diseases were consistent with the racial disparities in the rates of allergic diagnoses^20^(Figure S6C). Taken together, these data point to the insufficiency in the practice of adjusting for socioeconomic or demographic factors. Therefore, adjusting for socioeconomics without assessing regional differences in toxicant exposures may fail to properly assess the impact of social determinants on disease outcomes.

### P.A.D.D.L.E. offers a tool to assess toxicant associations with disease and demographics

**Fig. 4:**
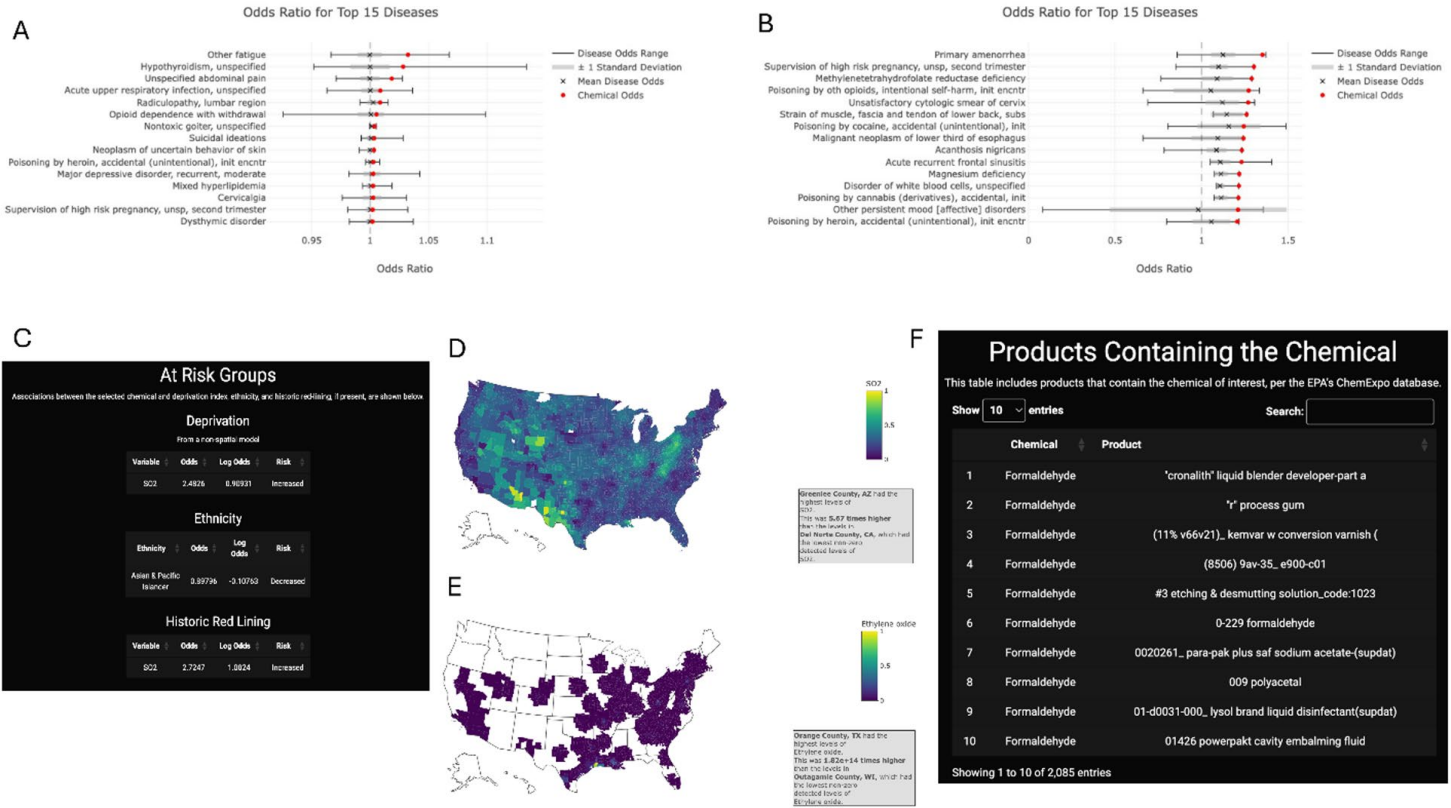
P.A.D.D.L.E. outputs when searching by chemical. (**A-B**) Nonspatial (**A**) and spatial correlations as odds ratios for diseases in adults age 18–54 (A) or 18 and older (**B**) linked with exposure to SO_2_. (**C**) Output from P.A.D.D.L.E. indicating the association between SO_2_ exposure and zip code deprivation, percent ethnicity for zip codes, and the historic redlining score. (**D**) Mapping of the average reported exposure of SO_2_ for years 2010–2016 for contrasting against 2016 clinical data. Scaled by minimum and maximum with imbedded details on the fold-change from the lowest non-zero zip code concentration to the highest value. (**E**) As in D but for ethylene oxide exposure. (**F**) Output for products known to contain formaldehyde based on EPA data.

To assist researchers in an investigation of chemical associations, we created an interactive webpage we termed the “Pollution Associated Disease Diagnosis Likelihood Estimator” (P.A.D.D.L.E.) [https://ratleyg-paddle.share.connect.posit.cloud/; and downloadable for running in R at http://github.com/ratleyge/PADDLE]. Users can search associations by chemical, diagnosis, or social determinant. Graphs for chemicals display the diseases associated with that chemical as a red dot to indicate the strength of the association against the range of all chemical associations for the diagnosis (Fig. 4A-B). As an example, nonspatial analysis in adults (18–54 years) for sulfur dioxide (SO_2_) was linked to fatigue and hypothyroidism (Fig. 4A); spatial analysis identified links between SO_2_ and female reproductive function among other disorders (Fig. 4B). Groups at risk of exposure include those in high deprivation zip codes (OR = 2.5) and historically redlined zip codes (OR = 2.7) (Fig. 4C). The only association between SO_2_ and racial demographics was a reduced risk of exposure in zip codes with a greater proportion of Asian and Pacific Islander populations (OR = 0.9; Fig. 4C). Mapping within the continental United States (CONUS) is available for each chemical and is min-max scaled (Fig. 4D). For chemicals like SO_2_, the most exposed of the CONUS have approximately 5-fold higher concentrations than the lowest non-zero exposed zip codes (Fig. 4D). In contrast, differences in exposure to the potent carcinogen ethylene oxide^35^reached 150-trillion-fold higher in Orange and Jefferson County, Texas (Fig. 4E) compared to the counties with the lowest non-zero levels. P.A.D.D.L.E. also searches the EPA database for commercial products known to contain the chemical. For SO_2_, no commercial product was reported; however, for a more common agent like formaldehyde, several products are identified (Fig. 4F).

### P.A.D.D.L.E. assesses disease associations with toxicants

**Fig. 5:**
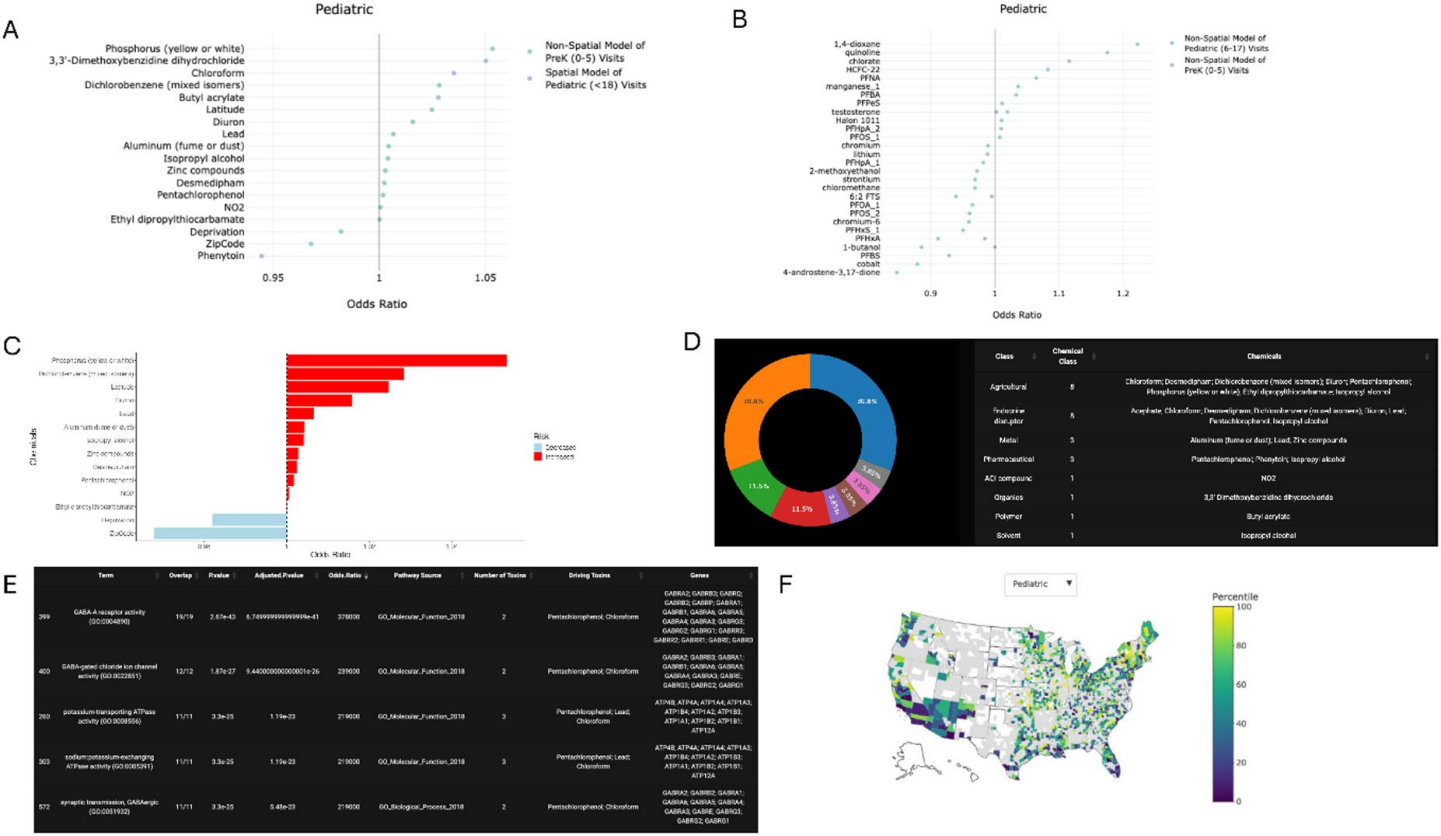
P.A.D.D.L.E. outputs when searching by diagnosis. (**A-B**) Nonspatial and spatial correlations as odds ratios for exposure to indicated toxicants for visit rate of ADHD in children age 0–5 years or 6–17 years for air pollution (**A**) and water pollution (**B**). (**C**) Output from P.A.D.D.L.E. indicating only the nonspatial associations with ADHD and air pollutants. (**D**) Output showing chemical classes of the toxicants associated with ADHD, combined type. Note overlapping definitions are possible so toxicants may be both agricultural products and endocrine disruptors. (**E**) Output indicating the pathway enrichment analysis. All toxicants linked to ADHD is contrasted against known protein-toxicant databases to extract the protein and/or genes impacted by the toxicants. Then, GO pathways are assessed to identify if the genes/proteins impacted by the toxicants associated with ADHD concentrate in any given pathway. (**F**) Mapping of the visit rates for ADHD in children 6–17 years of age for 2016 from AHRQ database. Rates are scaled to percentile. P.A.D.D.L.E. site output also displays the 10 counties with the highest visit rates for each age bracket.

In both spatial and nonspatial analyses of pediatric diseases, attention-deficit hyperactivity disorder (ADHD), combined type identified significant chemical associations (Fig. 1; Figure S1). Focusing on this example, P.A.D.D.L.E. identifies that the top associations include phosphorus, 3,3’-dimethoxybenzidine dihydrochloride, and chloroform for air pollution (Fig. 5A). Top water pollutant associations were 1,4-dioxane, quinoline, and chlorate (Fig. 5B). Users can use the overview of all relevant models or stratify by spatial/nonspatial and age bracket (Fig. 5C). Assessment of the overall impactors (air and water) by chemical class can be performed. For ADHD, such analysis indicates that most of the chemicals associated with diagnosis are used in agriculture (pesticides, herbicides, and/or insecticides) and/or are known endocrine disruptors (Fig. 5D). Pathway enrichment analysis can be performed by assessing the human signaling pathways which contain the known protein/gene targets impacted by the chemicals linked with the diagnosis (Fig. 5E). Consistent with clinical data^36^, such analysis for ADHD suggests an enrichment for GABA signaling targets in the chemicals linked to the disease (Fig. 5E). Similarly, assessment of Alzheimer’s disease reproduced prior associations^32,37,38^with PM_2.5_, PFAS compounds, and oxidative stress (Figure S7A-C). Mapping of disease rates is also possible (Fig. 5F), and the site outputs the 10 counties with the highest visit rates for the selected disease for each age bracket used in the nonspatial modeling. This feature may identify “hot spots” worthy of follow up investigation.

Contrasting our prior results for AD^11,12^ (which used private databases with clinic visits from 2017 to 2019) versus those using P.A.D.D.L.E. (which relies on the public AHRQ data from only 2016) we find that while the specific chemicals differ, the top associations were also in the BTEX family of chemicals (Figure S8A-C). Pathway analysis was also consistent with our previous reports and indicated cholinergic signaling, TRPA1, along with lipid and nitrogen metabolism (Figure S8D-E). Thus, while the specific top chemical associations varied, different clinical databases identified the same chemical class and mechanistic pathogenesis.

### P.A.D.D.L.E. assesses social factors associated with chemical exposures

It would be fallacious to directly connect chemical exposures with demographics at the zip code level because one cannot assume that the dominant group by population is also the most frequently exposed. However, assessing differences in potentially disease-causing toxicants may assist researchers in avoiding the paradigm of genetic determinism which assumes innate differences in populations meaningfully contribute to differences in disease outcomes^39,40^. As described in the overviews (Figs. 2 and 3), P.A.D.D.L.E. indicates a strong association between propiconazole and deprivation index (Figure S9A), NO_2_ and the percentage of Black Americans in a zip code (Figure S9B), heavy metals in the water and the percentage Hispanic in a zip code (Figure S9C), thiodicarb and the percent White population (Figure S9D), and historic redlining linked with potassium bromate in the air (Figure S9E) and PFHpA in the water (Figure S9F).

Referencing the diseases associated with the chemicals with disparate exposures offers a possibility to triangulate analysis between chemicals, diseases, and at-risk populations. As summarized in Supplemental Table 5, propiconazole was associated with deprivation and linked to early labor, and cervical and vaginal disorders in adults as well as pediatric hemangiomas, congenital malformations, feeding problems, and delayed puberty. Several chemicals disproportionately concentrated in Black zip codes were linked to scoliosis and other malformations of the spinal column which, similar to the racial disparities in the AQI compounds linked to allergic disease^34^, are consistent with reported disparities^41^. The heavy metals which are more common in zip codes with higher Hispanic populations are each linked to neurodevelopmental and behavioral disorders with reported disparities in disease rates^42^ and consistent with reported associations between the diagnoses and elevated blood levels of these heavy metals^43,44^. Among others, exposures more common among White populations which are congruent with racial disparities included tribenuron methyl’s link to Crohn’s disease^45^.

## Discussion

The correlation between non-communicable diseases and industrial lifestyles has become clear. Industrialization typically brings an increased risk of pollution, as well as enhanced exposure to processed foods^46^ and a reduction in parasitic exposures under the hygiene hypothesis^47^. An ideal database would be able to assess disease incidence against personal exposure to pollutants as well as incorporate dietary and occupational information. Given that the technology for person-level untargeted multi-pollutant detection does not yet exist, population-level data can only generate hypotheses to direct targeted and mechanistic inquires, similar to the discoveries of endocrine disruptors^48^.

However, while only hypothesis generating, the major limitation of this work is that the AHRQ database exclusively captured visits in the US in 2016. Although AHRQ’s database has the advantage of containing both inpatient and outpatient visit information, our models’ accuracy would greatly improve should the program collect additional years. Furthermore, alterations in the model parameters and assumptions might provide differing results; the visit rates and toxicant exposure data is either open source from US government sources or provided within our full code (see methods for download). Researchers may wish to run different models for their specific disease of interest prior to initiating mechanistic validation studies. Lack of longitudinal surveillance also precludes detection of delayed toxicant effects, such as the chemicals for which childhood exposure contributes to adult atopy^1^. Our prior work on AD used a commercial database included data from 2017 to 2019 identified slightly different specific chemicals, however, the fact that the same chemical class was identified speaks to the value of using pathway and chemical class aggregation in our assessments.

A similar limitation of our work is that it is limited to pollution released in the United States and do not capture exposures that may cause harm as commercial products unless the exposure also creates pathology in the areas surrounding their manufacturing point sources. Nations with centralized health records could however mirror our approach by collecting pollution data in their countries and/or product exposure surveys. Another limitation is the incongruency across the ChemExpo, GO, and Kegg databases used in assessing functional consequences of chemical exposure. For example, the database for known protein-pollutant interactions is only a subset of the overall pollutant data and is enriched for gene and protein targets that have received greater investigative attention. Previous work has also modeled connections between exposures and biologic pathways with greater resolution, but for a limited number of toxicants^49^. An additional approach to pathway identification proposed integrating exposures to single nucleotide polymorphism data^50^, however such approaches would be confounded by population stratification, particularly as it related to race and income. Therefore, the pathway analysis offered by P.A.D.D.L.E. may still serve to generate research hypotheses and researchers should evaluate their hypothesis through all available tools.

The authors stress that the data outputs from P.A.D.D.L.E. are correlations and associations only and should not be assumed to be causal, even when statistically significant. Any association identified should either be assessed against the established literature or be experimentally modeled before drawing any conclusions between the associations presented. For example, although negative associations could theoretically represent a protective effect of a given chemical, because our clinical data is derived from visits to healthcare providers rather than diagnosed individuals, it seems more likely that some chemicals may contribute to diseases which displace visits for other ailments. For instance, areas with the highest rates of COVID-19 infections saw a drop in outpatient visits for non-COVID related ailments^51^. We predict that toxicants like NO_2_ may similarly generate asthma exacerbations at rates which limit healthcare resources for other unrelated ailments. Using healthcare visits as the primary outcome also likely deflates the magnitude of our detected odds ratios. Unlike patient registries, which may capture any individual with the diagnosis, visit databases only capture those who sought medical attention during the period of observation.

However, with these caveats in mind, P.A.D.D.L.E. offers several advantages over traditional approaches. Assessing several toxicants simultaneously using penalized regression models may help researchers mitigate the risk of making false associations due to co-linearity of different toxicants. Furthermore, using a broader search which contrasts chemical classes versus disease pathways may inform mechanistic follow up studies to identify disease processes. For the associated exposures with mechanistic plausibility, this work will also allow researchers to adjust for expected harms from those exposures rather than simply relying on proxies like race or poverty. Finally, this work may help identify disease “hot spots” which may help investigators to identify regional factors. If the disease tracking program by AHRQ was conducted annually, such work could also serve as “real time monitoring” for chronic diseases in ways akin to those employed for communicable diseases.

While the presentation of numerous associations adds complexity to our report, it also provides valuable context. Chemical associations which are consistent across model types and age groups should take priority when selecting mechanistic evaluation follow up. Meanwhile, the associations which already have literature supported mechanistic connections should take priority in when selecting clinical mitigation studies. Thus, while potentially overwhelming, the volume of associations can help contextualize the strength of any specific disease-toxicant link of interest. Overall, P.A.D.D.L.E. offers a preliminary yet valuable framework to perform untargeted assessment of the environmental toxicants which may contribute to pathology.

## Supplementary Information

Below is the link to the electronic supplementary material.

Supplementary Material 1

## Data Availability

All code and files required to run PADDLE, including all data presented in this manuscript, can be directly downloaded from GitHub: [https://github.com/ratleyge/PADDLE]. Spreadsheets indicating the levels of toxicant exposures by disease/zip code are accessible in this repository. Due to privacy protections, absolute visitation rates for each disease by zip code are not publicly available but can be obtained through direct application to the AHRQ. Relative visit rates expressed as percentiles by disease and zip code are available in the GitHub repository under PADDLE/Data/Disease_mapping_data. Those seeking access to the SyH-DR database to establish raw visit numbers per zip code must send their requests to AHRQ. References for all packages used in the analysis and website design can be found in the supplement.

## Abbreviations

AD: Atopic dermatitis
ADHD: Attention deficient hyperactivity disorder
ADI: Area deprivation index
AHRQ: Agency for Healthcare Research and Quality
AQI: Air quality index
BTEX: Benzene, toluene, ethylbenzene, and xylene family chemicals
ChemExpo: Chemical exposure
CMS: Center for medicare services
CO: Carbon dioxide
CONUS: Continental United States
EPA: Environmental protection agency
GO: Gene ontology
HBCD: Hexabromocyclododecane
HRS: Historic redlining score
ICD: International classification of disease
KEGG: Kyoto encyclopedia of genes and genomes
NO2: Nitric dioxide
OR: Odds ratio
PFAS: Per- and polyfluoroalkyl substances
PFBS: Perfluorobutanesulfonic acid
PFHpA: Perfluoroheptanoic acid
PM2.5: Particulate matter less than 2.5 microns
PM10: Particulate matter less than 10 microns
RSEI: Risk-screening environmental indicators
SO2: Sulfur dioxide
SyH-DR: Synthetic healthcare database for research
TRI: Toxic release inventory
TRPA1: Transient receptor potential Ankyrin 1
UCMR: Unregulated contaminant monitoring rule
