# Supplementary material for: P.A.D.D.L.E.: a hypothesis generation tool for assessing pollution’s potential role in disease
Source: Sci Rep. 2026 Feb 13;16:8808. doi: 10.1038/s41598-026-39836-2 (PMC12982754; doi:10.1038/s41598-026-39836-2)
Supplement: Supplementary file 1 — Supplementary Material 1 [file 41598_2026_39836_MOESM1_ESM.docx]

**Supplemental Materials:**

**P.A.D.D.L.E. : A hypothesis generation tool for assessing pollution’s potential role in disease**

Grace Ratley^1,2^, Aditi Vijendra^1+^, Jalin Jordan^1+^, Pranav Thota^1^, Jordan Zeldin^1^, Prem Prashant Chaudhary^1^, Ian A Myles^1,*^

^1^ Epithelial Therapeutics Unit, National Institute of Allergy and Infectious Disease, National Institutes of Health, Bethesda, MD, USA

^2^ Unit of Integrative Metabolomics, Institute of Environmental Medicine Karolinska Institute, Stockholm, Sweden.

+ denotes equal contribution

*Corresponding author: [mylesi@niaid.nih.gov](mailto:mylesi@niaid.nih.gov)

**Supplemental Table 1**

|  | **0–5-year-olds** | | | **6-17-year-olds** | | |
| --- | --- | --- | --- | --- | --- | --- |
| **ICD** | **Top Assoc** | **OR** | **Source** | **Top Assoc** | **OR** | **Source** |
| **Behavioral Health/CNS Disorders** | | | | | | |
| Unspecified speech disturbances | 2,4-Dinitrophenol | 1.09 | Air | 2,4-Dinitrophenol | 1.08 | Air |
| Anxiety disorder, unspecified | Chlorsulfuron | 1.21 | Air | Captan | 1.13 | Air |
| Attention-deficit hyperactivity disorder, combined type | Phosphorus (yellow or white) | 1.05 | Air | Potassium bromate | 1.26 | Air |
| Attention-deficit hyperactivity disorder, unspecified type | Chlorsulfuron | 1.14 | Air | Chlorimuron ethyl | 1.28 | Air |
| Autistic disorder | Triethylamine | 1.10 | Air | 4,4'-Methylenedianiline | 1.28 | Air |
| Disruptive mood dysregulation disorder | chlorate | 1.00 | Water | chromium-6 | 1.12 | Water |
| Major depressive disorder, recurrent, unspecified | Propargyl alcohol | 1.02 | Air | Chromium | 1.01 | Air |
| Major depressv disorder, recurrent severe w/o psych features | Vanadium | 1.01 | Air | chlorate | 1.01 | Water |
| Mixed receptive-expressive language disorder | Mecoprop | 1.09 | Air | Population_Density | 1.10 | Air |
| Muscle weakness (generalized) | Cyanide compounds | 1.08 | Air | Piperonyl butoxide | 1.21 | Air |
| Other speech disturbances | 2,4-Dinitrophenol | 1.06 | Air | Cyanide compounds | 1.06 | Air |
| Post-traumatic stress disorder, unspecified | Carbon disulfide | 1.02 | Air | PFPeS | 1.18 | Water |
| Unspecified conjunctivitis | PFPeA | 1.05 | Water | 1-butanol | 1.01 | Water |
| Hypermetropia, bilateral | Benzoyl chloride | 1.01 | Air | Vinyl acetate | 1.10 | Air |
| **Infection** | | | | | | |
| Infectious gastroenteritis and colitis, unspecified | Diglycidyl resorcinol ether | 1.03 | Air | Diglycidyl resorcinol ether | 1.10 | Air |
| Acute nasopharyngitis [common cold] | tert-Butyl alcohol | 1.51 | Air | tert-Butyl alcohol | 1.28 | Air |
| Acute pharyngitis, unspecified | Tetrabromobisphenol-A (TBBPA) | 1.26 | Air | 1,3-Dichloro-1,1,2,2,3-pentafluoropropane | 1.24 | Air |
| Acute upper respiratory infection, unspecified | Potassium bromate | 1.33 | Air | Propoxur | 1.15 | Air |
| Dental caries, unspecified | Asbestos (friable) | 1.07 | Air | O3 | 1.12 | Air |
| Fever, unspecified | cobalt | 1.18 | Water | Fluorine | 1.08 | Air |
| Impetigo, unspecified | PFPeA | 1.02 | Water | PFPeS | 1.02 | Water |
| Otitis media, unspecified, left ear | Sodium dimethyldithiocarbamate | 1.05 | Air | 2-methoxyethanol | 1.03 | Water |
| Urinary tract infection, site not specified | 1-butanol | 1.05 | Water | chloromethane | 1.08 | Water |
| Viral infection, unspecified | Potassium bromate | 1.15 | Air | Oryzalin | 1.12 | Air |
| **Gastrointestinal Disorders** | | | | | | |
| Diarrhea, unspecified | cobalt | 1.05 | Water | 2-Nitropropane | 1.00 | Air |
| Nausea with vomiting, unspecified | cobalt | 1.04 | Water | Tetrabromobisphenol-A (TBBPA) | 1.15 | Air |
| Noninfective gastroenteritis and colitis, unspecified | Potassium bromate | 1.05 | Air | chlorate | 1.11 | Water |
| Unspecified abdominal pain | Bromoxynil | 1.04 | Air | cobalt | 1.13 | Water |
| Vomiting, unspecified | cobalt | 1.05 | Water | 2-Nitropropane | 1.07 | Air |
| **Other** | | | | | | |
| Cough | Deprivation | 1.11 | Air | Methyl isobutyl ketone | 1.06 | Air |
| Other specified disorders of Eustachian tube, bilateral | Creosote, coal tar | 1.04 | Air | vanadium | 1.00 | Water |
| Unspecified asthma, uncomplicated | 2,2-Dichloro-1,1,1-trifluoroethane | 1.10 | Air | Zinc compounds | 1.22 | Air |

**Supplemental Table 1: Top correlations in nonspatial analysis of adults over 18 years of age versus air and/or water pollution.** Overall associations for disease diagnosis rates and top pollutants per age bracket were identified and sorted by organ system. Strongest associations by odds ratio across the population (OR) for each disorder are presented for pre-K (0-5 years of age) and pediatric (6-17 years) age brackets.

**Supplemental Table 2**

| **ICD** | **Top Association** | **Odds Ratio** |
| --- | --- | --- |
| **Behavioral Health/CNS Disorders** | | |
| Adjustment disorder with mixed anxiety and depressed mood | Potassium bromate | 1.16 |
| Attn-defct hyperactivity disorder, predom inattentive type | Parathion | 1.1472 |
| Paranoid schizophrenia | 4,4'-Methylenedianiline | 1.3871 |
| Mild intellectual disabilities | Potassium bromate | 1.1593 |
| Myopia, bilateral | 3-Iodo-2-propynyl butylcarbamate | 1.1523 |
| Myopia, unspecified eye | 4,4'-Methylenedianiline | 1.3371 |
| Specific developmental disorder of motor function | Parathion | 1.2014 |
| Other psychoactive substance abuse, uncomplicated | Parathion | 1.149 |
| Transient alteration of awareness | 4,4'-Methylenedianiline | 1.2235 |
| Unspecified acute conjunctivitis, unspecified eye | 4,4'-Methylenedianiline | 1.2123 |
| **Maternal, Reproductive, and Early Childhood Development** | | |
| Acute vaginitis | 4,4'-Methylenedianiline | 1.1538 |
| Congen malform syndromes predom affecting facial appearance | Dimethyl phthalate | 1.1667 |
| Congenital deformity of spine | NO2 | 1.1568 |
| Pervasive developmental disorder, unspecified | Benomyl | 1.3536 |
| Renal dysplasia | PM2.5 | 1.158 |
| Neonatal difficulty in feeding at breast | Dimethyl phthalate | 1.1692 |
| Unspecified cleft palate with unilateral cleft lip | PM10 | 1.1468 |
| Other specified noninflammatory disorders of vagina | 4,4'-Methylenedianiline | 1.1833 |
| **Infection** | | |
| Bacteremia | Parathion | 1.1721 |
| Cutaneous abscess of left upper limb | Tetrabromobisphenol-A (TBBPA) | 1.2082 |
| Cutaneous abscess of right foot | Benomyl | 1.232 |
| Cutaneous abscess of right upper limb | Parathion | 1.1642 |
| Suppurative otitis media, unspecified, right ear | Parathion | 1.1746 |
| Other chronic diseases of tonsils and adenoids | Fluorouracil (5-fluorouracil) | 1.4087 |
| Other chronic diseases of tonsils and adenoids | Oryzalin | 1.4461 |
| **Cardiovascular** | | |
| Other specified cardiac arrhythmias | Benomyl | 1.2537 |
| Other cardiomyopathies | CO | 1.1488 |
| Secondary hypertension, unspecified | NO2 | 1.1732 |
| **Physical Injury** | | |
| Oth injuries of left wrist, hand and finger(s), init encntr | Tetrabromobisphenol-A (TBBPA) | 1.2525 |
| Oth tear of medial meniscus, current injury, r knee, init | Deprivation | 1.1485 |
| Unspecified injury of left lower leg, initial encounter | Tetrabromobisphenol-A (TBBPA) | 1.1597 |
| Unspecified injury of right foot, initial encounter | Fluorouracil (5-fluorouracil) | 1.326 |
| Unspecified sprain of right shoulder joint, init encntr | Propoxur | 1.5899 |
| **Hematology** | | |
| Abnormal finding of blood chemistry, unspecified | Deprivation | 1.1468 |
| Antineoplastic chemotherapy induced pancytopenia | CO | 1.1807 |
| Sickle-cell disease without crisis | 1-Bromopropane | 1.1702 |
| Sickle-cell trait | Thiram | 1.1791 |
| Iron deficiency | Methoxychlor | 1.2565 |
| Iron deficiency anemia secondary to blood loss (chronic) | 4,4'-Methylenedianiline | 1.3162 |
| Other specified abnormal findings of blood chemistry | Benomyl | 1.1467 |

**Supplemental Table 2: Top correlations in spatial analysis of children under 18 years of age versus air pollution.** Overall associations for disease diagnosis rates and air pollutants were assessed. The top odds ratios were identified and sorted by organ system.

**Supplemental Table 3**

|  | 18-54 years | | 55-74 years | | 75+ years | |
| --- | --- | --- | --- | --- | --- | --- |
| ICD | Top Assoc | OR | Top Assoc | OR | Top Assoc | OR |
| **Behavioral Health/CNS Disorders** | | | | | | |
| Major depressive disorder, single episode, unspecified | PM2.5 | 1.06 | Tetrachloroethylene (Perchloroethylene) | 1.04 | Methylene bromide | 1.05 |
| Mental disorder, not otherwise specified | PFHxA (water) | 1.09 | manganese (water) | 1.01 | 2,4-Dinitrophenol | 1.01 |
| Muscle weakness (generalized) | p-Dinitrobenzene | 1.10 | p-Dinitrobenzene | 1.16 | 2,2-Dichloro-1,1,1-trifluoroethane | 1.06 |
| Nicotine dependence, cigarettes, uncomplicated | Mercury | 1.08 | Toluene | 1.06 | Zinc | 1.03 |
| Opioid dependence with withdrawal | tert-Butyl alcohol | 1.10 | Potassium bromate | 1.02 | Potassium bromate | 1.02 |
| Opioid dependence, in remission | Piperonyl butoxide | 1.09 | Vinyl fluoride | 1.00 | Vinyl fluoride | 1.00 |
| Opioid dependence, uncomplicated | Potassium bromate | 1.56 | Piperonyl butoxide | 1.10 | Boron trichloride | 1.00 |
| Other abnormalities of gait and mobility | Vanadium | 1.00 | Thiobencarb | 1.06 | Thiobencarb | 1.06 |
| Other chronic pain | Chlorimuron ethyl | 1.08 | Aniline | 1.02 | Aniline | 1.02 |
| Schizophrenia, unspecified | Potassium bromate | 1.06 | Potassium bromate | 1.02 | Potassium bromate | 1.02 |
| Unsp dementia, unsp severity, without beh/psych/mood/anx | Acrolein | 1.02 | Dimethoate | 1.00 | Di(2-ethylhexyl) phthalate | 1.05 |
| **Endocrine** | | | | | | |
| Age-related osteoporosis w/o current pathological fracture | p-Cresidine | 1.01 | Myclobutanil | 1.02 | CO | 1.05 |
| Hyperlipidemia, unspecified | Mercury compounds | 1.03 | Mercury compounds | 1.06 | Cumene | 1.10 |
| Hypothyroidism, unspecified | PM2.5 | 1.04 | Dibenzofuran | 1.06 | O3 | 1.06 |
| Other spondylosis with myelopathy, lumbar region | Propargyl alcohol | 1.04 | o-Dinitrobenzene | 1.13 | o-Dinitrobenzene | 1.13 |
| Type 2 diabetes mellitus without complications | Potassium bromate | 1.02 | cobalt (water) | 1.09 | o-Toluidine | 1.09 |
| Vitamin D deficiency, unspecified | 1,1-Dimethyl Hydrazine | 1.49 | Sodium azide | 1.29 | 1,2-Dichloroethylene | 1.08 |
| **Cardio-Renal Disorders** | | | | | | |
| Anemia in chronic kidney disease | Cresol (mixed isomers) | 1.04 | 1,1,1,2-Tetrachloro-2-fluoroethane (HCFC-121a) | 1.01 | 1,2-Dichloroethylene | 1.04 |
| Athscl heart disease of native coronary artery w/o ang pctrs | Calcium cyanamide | 1.00 | PM2.5 | 1.05 | Nonylphenol | 1.05 |
| Chest pain, unspecified | PM10 | 1.02 | Propargyl alcohol | 1.08 | Propargyl alcohol | 1.08 |
| Essential (primary) hypertension | PM2.5 | 1.24 | Potassium bromate | 1.13 | Propargyl alcohol | 1.12 |
| Hypertensive chronic kidney disease w stg 1-4/unsp chr kdny | p-Cresidine | 1.07 | Merphos | 1.06 | PM2.5 | 1.06 |
| **Gastrointestinal Disorders** | | | | | | |
| Dvrtclos of lg int w/o perforation or abscess w/o bleeding | PM10 | 1.03 | Phosphorus (yellow or white) | 1.08 | Phosphorus (yellow or white) | 1.08 |
| Gastro-esophageal reflux disease without esophagitis | PM2.5 | 1.07 | chloromethane (water) | 1.09 | Bromochlorodifluoromethane | 1.31 |
| Other hemorrhoids | Desmedipham | 1.00 | Propylene oxide | 1.09 | Propylene oxide | 1.09 |
| **Pulmonary** | | | | | | |
| Chronic obstructive pulmonary disease, unspecified | 2,4-Dinitrophenol | 1.04 | Aluminum phosphide | 1.04 | Benzoyl chloride | 1.05 |
| Shortness of breath | Triphenyltin hydroxide | 1.01 | 1,1,1,2-Tetrachloro-2-fluoroethane (HCFC-121a) | 1.02 | Bis(2-chloroethoxy)methane | 1.05 |
| Unspecified asthma, uncomplicated | NO2 | 1.13 | Creosote, coal tar | 1.03 | Creosote, coal tar | 1.03 |
| **Infection** | | | | | | |
| Cellulitis of trunk, unspecified | Oryzalin | 1.10 | Tetracycline hydrochloride | 1.00 | Tetracycline hydrochloride | 1.00 |
| Human immunodeficiency virus [HIV] disease | Potassium bromate | 1.08 | Creosote, coal tar | 1.03 | Potassium bromate | 1.04 |
| **Other** | | | | | | |
| Anemia due to antineoplastic chemotherapy | Ethylene glycol | 1.00 | Oryzalin | 1.11 | Oryzalin | 1.11 |
| Other seborrheic keratosis | Diglycidyl resorcinol ether | 1.02 | Lactofen | 1.09 | Lactofen | 1.09 |

**Supplemental Table 4: Top correlations in nonspatial analysis of adults over 18 years of age versus air and/or water pollution.** Overall associations for disease diagnosis rates and top 15 pollutants per age bracket were identified and sorted by organ system and odds ratio across the population (OR). Pollutant source was air unless specified as water.

**Supplemental Table 4**

| **ICD** | **Top Association** | **Odds Ratio** |
| --- | --- | --- |
| **Maternal, Reproductive, and Early Childhood Development** | | |
| Inapprop chg quantitav hCG in early pregnancy | 1,2,3-Trichloropropane | 1.474407678 |
| Inapprop chg quantitav hCG in early pregnancy | 1,4-Dichloro-2-butene | 1.404205066 |
| Inapprop chg quantitav hCG in early pregnancy | N-Nitrosodiphenylamine | 1.414987747 |
| Malignant neoplasm of ovrlp sites of unsp female breast | CO | 1.418824466 |
| Trichomonal vulvovaginitis | Deprivation | 1.62407067 |
| Trichomoniasis, unspecified | Deprivation | 1.741389285 |
| Intraductal carcinoma in situ of unspecified breast | NO2 | 1.448216685 |
| Liver and biliary tract disord in pregnancy, third trimester | NO2 | 1.469459389 |
| Malignant neoplasm of ovrlp sites of unsp female breast | NO2 | 1.483388992 |
| Maternal care for benign tumor of corpus uteri, first tri | NO2 | 1.435771727 |
| Secondary and unsp malignant neoplasm of intrapelv nodes | NO2 | 1.409954836 |
| Submucous leiomyoma of uterus | NO2 | 1.411666446 |
| Supervision of elderly multigravida, second trimester | NO2 | 1.634861847 |
| Supervision of elderly multigravida, unspecified trimester | NO2 | 1.460740102 |
| Neonatal jaundice, unspecified | Propargyl alcohol | 1.494234763 |
| Unspecified diabetes mellitus in pregnancy, third trimester | NO2 | 1.422969493 |
| **Behavioral Health/CNS Disorders** | | |
| Opioid dependence, in remission | 1,2,3-Trichloropropane | 1.737756731 |
| Blood alcohol level of 240 mg/100 ml or more | CO | 1.637757794 |
| Blood alcohol level of 240 mg/100 ml or more | NO2 | 1.509436296 |
| Blood alcohol level of 240 mg/100 ml or more | O3 | 1.733334913 |
| Cocaine abuse, uncomplicated | NO2 | 1.405848372 |
| Cocaine dependence, uncomplicated | NO2 | 1.41671701 |
| Mild intellectual disabilities | Deprivation | 1.431309147 |
| Sedative, hypnotic or anxiolytic dependence, uncomplicated | NO2 | 1.511986244 |
| Impulse disorder, unspecified | Propargyl alcohol | 1.399282551 |
| Cannabis dependence, in remission | Potassium bromate | 1.48449395 |
| Cannabis dependence, in remission | Silver | 1.422998696 |
| Coma scale, best verbal response, oriented, EMR | PM2.5 | 1.464304 |
| **Infection** | | |
| Human immunodeficiency virus [HIV] disease | NO2 | 1.521816859 |
| Non-prs chr ulcer of right heel and midft lmt to brkdwn skin | Deprivation | 1.413958695 |
| Acute recurrent frontal sinusitis | Deprivation | 1.406593315 |
| Syphilis, unspecified | CO | 1.496661324 |
| Syphilis, unspecified | NO2 | 1.673212371 |
| Unspecified blepharitis left lower eyelid | NO2 | 1.513924797 |
| Infection of amputation stump, right lower extremity | Pentachlorophenol | 1.535396123 |
| **Physical Injury** | | |
| Oth cause of strike by thrown, projected or fall obj, init | O3 | 1.454754295 |
| **Hematology** | | |
| Mantle cell lymphoma, unspecified site | Population Density | 1.498709651 |
| **Gastrointestinal** | | |
| Acute cholecystitis with chronic cholecystitis | Deprivation | 1.502959161 |
| Malignant neoplasm of anal canal | Piperonyl butoxide | 1.414435288 |
| Secondary malignant neoplasm of small intestine | NO2 | 1.437019204 |
| Dental caries, unspecified | Deprivation | 1.511509534 |
| **Monogenic Disorders** | | |
| Cystic fibrosis with pulmonary manifestations | Crotonaldehyde | 1.404223594 |
| Sickle-cell disease without crisis | Deprivation | 1.442111428 |
| Hb-SS disease with crisis, unspecified | NO2 | 1.540357586 |
| Sickle-cell trait | PM2.5 | 1.417310703 |
| Sickle-cell trait | NO2 | 1.465317893 |

**Supplemental Table 4: Top correlations in spatial analysis of adults over 18 years of age versus air pollution.** Overall associations for disease diagnosis rates and air pollutants were assessed. The top odds ratios were identified and sorted by organ system.

**Supplemental Table 5**

| **Toxin** | **Social determinant risk factor** | **Disease associations** | **Disparities in diseases for at-risk groups** |
| --- | --- | --- | --- |
| Propiconazole | Deprivation | - Early labor, - Cervical disorders - Vaginal disorders - Hemangiomas - Congenital malformations - Feeding problems - Delayed puberty | Unknown |
| bromochlorodifluoromethane |  | - STEMI - Unspecified immunodeficiency |  |
| Cyfluthrin | % of self-reported Black population | - Scoliosis - Other spinal column abnormalities | Yes |
| Hexazinone |  |  |  |
| 2,4-dichlorophenoxy acetic acid |  |  |  |
| NO_2_ |  | - Asthma | Yes |
| CO |  |  |  |
| Vanadium | % of self-reported Hispanic population | - Autism - Dysphagia - Gait abnormalities - Major depressive disorder (MDD) - Type 2 diabetes | Yes |
| Strontium |  | - Feeding difficulties - Epilepsy - MDD |  |
| Lithium |  | - Epilepsy - Hearing loss - MDD - Affective mood disorder - Bipolar disorder - Schizophrenia - Type 2 diabetes |  |
| Ethoprop | % of self-reported White population | - Chronic pain syndrome | Yes |
| Tribenuron methyl |  | - Crohn’s Disease |  |
| PFHpA | Historic redlining scores | - Disorders of male reproductive organs - Colon cancer | Unknown |

**Supplemental Table 5:** Summary of assessments of chemicals that show disparities in exposure by social determinant, the diseases linked to these chemicals by P.A.D.D.L.E. and whether those disease have known disparities in disease rates for the at-risk group.

**Supplemental Figures:**

**Supplemental Figure 1: Associations between air pollution and diseases in children aged 0-5 years**. Non-spatial correlations displayed as beta coefficients (β) for diagnosis rate for children aged 0-5 years by zip code. A mean value was derived for the coefficients between every disease and every variable across the entire cohort. Diseases containing coefficients that were greater than 5 standard deviations removed from the mean are displayed solely to aide in readability. All associations are included on the website.

**Supplemental Figure 2: Associations between air pollution and diseases in adults aged 18-54 years**. Non-spatial correlations displayed as beta coefficients (b) for diagnosis rate for adults aged 18-54 years by zip code. A mean value was derived for the coefficients between every disease and every variable across the entire cohort. Diseases containing coefficients that were greater than 5 standard deviations removed from the mean are displayed solely to aide in readability. All associations are included on the website.

**Supplemental Figure 3: Associations between water pollution and diseases in adults aged 18-54 years**. (A) Non-spatial correlations for water pollution and diagnosis rate for adults aged 18-54 years by zip code displayed as odds ratio (OR). A mean value was derived for the OR between every disease and every variable across the entire cohort. Diseases containing OR that were greater than 5 standard deviations removed from the mean are displayed. (B) Difference between the percent of total diagnostic codes appearing among those with coefficients >5SD from mean as described in methods. Diseases containing OR that were greater than 5 standard deviations removed from the mean are displayed solely to aide in readability. All associations are included on the website. Significance calculated by Chi-squared for observed versus expected number of times a disease with the diagnostic code was identified among top hits. * = p <0.05; *** = p < 0.001; **** = p < 0.0001. Significance identified for adult E codes (Endocrine, Nutritional, Metabolic), adult and pediatric F codes (Mental and Behavioral Disorders), and adult and pediatric T codes (Injury, poisoning, other consequences of external causes).

**Supplemental Figure 4: Associations between air pollution and cancer diagnoses**. Non-spatial correlations for air pollution and cancer diagnosis rate for (A) children aged 6-17 years and (B) adults aged 18-54 years by zip code displayed as odds ratio (OR). A mean value was derived for the OR between every disease and every variable across the entire cohort. Diseases containing OR that were greater than 5 standard deviations for pediatric and 12 standard deviations for adults removed from the mean are displayed solely for readability. All correlations are found on the website.

**Supplemental Figure 5. Associations between percentage of population of self-reported ethnicity and chemical exposures.** (A) Nonspatial correlations for *a priori* selected variables expected to have disparate rates, displayed as beta coefficients (β) for chemical exposures from air pollution sources reported from 2013-2019 versus the percentage of the population with the self-described ethnicity in the 2020 census. (B) Spatial correlations displayed as odds ratio (OR) for chemical exposures from air and water pollution sources.

**Supplemental Figure 6: Associations between historic redlining score and air and water pollution by zip code**. (A) Mapped concentrations for indicated toxins which represent the three chemicals with the strongest associations with historic redlining scores from 2020 (HRS2020), indicated as mcg/m^3^ weighted by 30-mile catchment areas. (B) Nonspatial correlations for air and water pollution versus the zip code HRS202 displayed as odds ratio (OR). A mean value was derived for the OR between every disease and every variable across the entire cohort. Diseases containing OR that were greater than 2 standard deviations removed from the mean are displayed. (C) Nonspatial correlations for *a priori* selected air pollutant variables expected to impact atopic dermatitis and allergic disease.

**Supplemental Figure 7. P.A.D.D.L.E. outputs when searching for Alzheimer’s disease.** (A-B) Combined spatial and nonspatial correlation for the diagnosis of Alzheimer’s disease for air pollutants (A) and water pollutants (B). (C) GO pathways enriched for genes/proteins impacted by the toxins associated.

**Supplemental Figure 8. P.A.D.D.L.E. outputs when searching for atopic dermatitis.** (A-B) Nonspatial (A) and spatial (B) correlations as odds ratios for exposure to indicated air pollutants for visit rate of atopic dermatitis (AD) in children aged 0-5 years (A) or under 18 years (B). (C) Nonspatial analysis for correlations between water pollutants and AD in children 0-5 years of age. (D) Output showing chemical classes of the toxins associated with AD. (E) GO pathways enriched for genes/proteins impacted by the toxins associated with AD.

**Supplemental Figure 9. P.A.D.D.L.E. outputs when searching by social determinant.** (A) Nonspatial association between indicated air and/or water pollutants and deprivation index. (B) Spatial association between air pollutants and the percentage of the population within a zip code that identified as Black/African American on the 2020 US Census. (C) Nonspatial analysis for association between water pollutants and the percentage of the population within a zip code that identified as Hispanic on the 2020 US Census. (D) Nonspatial association between air pollutants and the percentage of the population within a zip code that identified as White. (E-F) Nonspatial association between indicated air (E) or water (F) pollutants and historic redlining score.

**Supplemental File**: Model code. The R script code provided is only related to running the models.  It does not provide the needed steps for data processing, intake, etc.  It is provided in order to show the parameters for the models used to generate the coefficients and odds ratios in the study.

**Nonspatial Model Code**

# Read and clean the chemicals data frame

# This data frame also contains covariates: census age distribution, deprivation index, latitude, longitude, and population density

#Pipe x into the function the makes it a matrix- with rows and columns, then keeps the row names, then makes everything a number

chemicals <- chemicals %>%

mutate(ZipCode = sprintf("%05d", ZipCode)) %>%

group_by(ZipCode) %>%

summarise(across(where(is.numeric), ~ sum(.x, na.rm = TRUE))) %>%

ungroup() %>%

filter(ZipCode %in% intersect(ZipCode, rates$ZipCode)) %>%

column_to_rownames("ZipCode") %>%

as.matrix(., rownames = TRUE)

chemicals <- chemicals[,-which(colSums(chemicals) == 0), drop = FALSE]

chemicals <- scale(chemicals)

# scale x (eleastic net requires gaussian distribution, scale makes it so)

rates[is.na(rates)] <- 0

rates <- rates %>%

column_to_rownames("ZipCode")

rates <- rates[rownames(chemicals),]

# Make a dataframe where all the rows are the variables in the x matrix remove zipcoed if it is in there

outputDf <- data.frame(Variable = c("(Intercept)", colnames(chemicals)))

modelAccuracy <- as.data.frame(matrix(nrow = 0, ncol = 3))

names(modelAccuracy) <- c("Disease", "RMSE", "R2")

set.seed(123) # For reproducibility

#start the for loop, this means start at second column of diseaseDF, go until the end

for (column in 3) {

#takes the current column under analysis and assigned it to the variable rates

ratesColumnTitle <- names(rates)[column]

# filter the rows of rates so the length of y and x are the same

y <- rates[rownames(chemicals),]

# y is the column that would go against the analysis.

y <- as.data.frame(y[, column, drop = FALSE])

# Fit Elastic Net model

# alpha = 0.5 for Elastic Net (0 = Ridge, 1 = Lasso)

elastic_net_model <- cv.glmnet(chemicals[rownames(y),], y[, 1], alpha = 0.5, standardize = TRUE)

# Plot cross-validated error

plot(elastic_net_model)

# Extract the best lambda

best_lambda <- elastic_net_model$lambda.min

cat("Best lambda:", best_lambda, "\n")

# Fit final model with the best lambda

final_model <- glmnet(chemicals[rownames(y),], y[, 1], alpha = 0.5, lambda = best_lambda, standardize = TRUE)

coef <- coef(final_model, s = "lambda.min")

coefs <- data.frame(Variable = coef@Dimnames[[1]][coef@i + 1], coefficient = coef@x)

# The output data from this analysis should be a data frame with 2 rows

names(coefs) <- c("Variable", ratesColumnTitle)

outputDf <- merge(outputDf, coefs, by = "Variable", all = TRUE)

}

#Write the final csv

write.csv(outputDf, "Disease coefficents.csv", row.names = FALSE)

**Spatial Model Code**

# Construct inverse distance-weighted spatial weights for Moran's I calculation

# Convert coordinates to spatial points object

lonlat <- cbind(Data$Longitude, Data$Latitude)

pts <- SpatialPoints(lonlat)

crdref <- CRS('+proj=longlat +datum=NAD83')

pts <- SpatialPoints(lonlat, proj4string = crdref)

# Create neighbor structure: all ZIP codes within 50 miles

nb_dist <- dnearneigh(x = pts, d1 = 0, d2 = 50 * 1.609344) # Convert miles to km

# Convert to inverse distance-weighted list

lw <- nb2listwdist(

neighbours = nb_dist,

x = pts,

type = "idw", # Inverse distance weighting

style = "W", # Row-standardized weights

alpha = 1, # IDW power parameter

zero.policy = TRUE

)

# Generate 4-level hierarchical clustering based on geographic distance

# Calculate great-circle distance matrix

mdist <- geosphere::distm(cbind(Data$Longitude, Data$Latitude))

# Hierarchical clustering with complete linkage

hc <- hclust(as.dist(mdist), method = "complete")

# Create nested cluster levels

Data$cluster0 <- as.factor(cutree(hc, k = round(nrow(Data) / 81))) # ~81 ZIPs/cluster

Data$cluster1 <- as.factor(cutree(hc, k = round(nrow(Data) / 27))) # ~27 ZIPs/cluster

Data$cluster2 <- as.factor(cutree(hc, k = round(nrow(Data) / 9))) # ~9 ZIPs/cluster

Data$cluster3 <- as.factor(cutree(hc, k = round(nrow(Data) / 3))) # ~3 ZIPs/cluster

# Z-score all numeric columns except the offset (Total)

# Identify numeric columns for scaling

numeric_cols <- sapply(Data, is.numeric)

# Exclude the offset variable from scaling

total_index <- which(colnames(Data) == "Total")

numeric_cols[total_index] <- FALSE

# Scale numeric columns (mean = 0, SD = 1)

scaled_matrix <- as.data.frame(lapply(Data[, numeric_cols], scale))

colnames(scaled_matrix) <- names(Data)[numeric_cols]

# Combine scaled numeric columns with non-numeric columns

Data <- cbind(Data[!numeric_cols], scaled_matrix)

# For each disease (ICD code), fit univariate models for each pollutant

Mixed_Effect <- function(ICD) {

start_time <- Sys.time()

# Extract disease-specific outcome (visit counts)

numerator <- disease_df[[ICD]]

# Define covariate list (everything except focal pollutant)

Covariates <- c(

Ages_list,

"Latitude",

# "Deprivation",

"Population_Density",

"cluster0", "cluster1",

"cluster2", "cluster3",

"Total"

)

# Parallel loop over all pollutants

MM_loop <- foreach(

i = 1:ncol(Data),

.combine = "rbind",

.errorhandling = 'remove'

) %dopar% {

# Skip if column is a covariate or cluster variable

if (colnames(Data)[i] %in% c("Total", "cluster0", "cluster1", "cluster2", "cluster3")) {

return(NULL)

}

tryCatch({

# Create pollutant-specific dataset

if (colnames(Data)[i] %in% Covariates) {

# If focal variable is a covariate, exclude it and other covariates

skips <- which(colnames(Data) %in% Covariates)

} else {

# Include focal pollutant + all covariates

skips <- which(colnames(Data) %in% c(colnames(Data)[i], Covariates))

}

Data_sample <- Data[, skips]

Data_sample <- Data_sample %>% relocate(as.name(colnames(Data)[i]))

# Fit negative binomial GLMM with nested spatial random effects

model <- glmer.nb(

numerator ~ .

- Total # Remove offset from fixed effects to add as offset

- cluster0 - cluster1 - cluster2 - cluster3 # Remove clusters to add as random effects

+ offset(log(Total)) # Add as offset

+ (1 | cluster0 / cluster1 / cluster2 / cluster3), # Nested random effects

data = Data_sample,

nAGQ = 0, # Laplace approximation

control = glmerControl(

optimizer = "bobyqa",

optCtrl = list(maxfun = 1e9)

)

)

# Extract model summary

summo <- summary(model)

# Calculate Moran's I for suggestive associations (p < 0.1)

if (summo$coefficients[2, 4] < 0.1) {

I_ac <- moran.test(

residuals(model, type = "pearson"),

lw,

zero.policy = TRUE

)

MI <- I_ac$estimate[1]

MI_p <- I_ac$p.value

} else {

MI <- NA

MI_p <- NA

}

# Check overdispersion

overdisp_output <- capture.output(RVAideMemoire::overdisp.glmer(model))

ratio <- sub(".*ratio: ([0-9.]+).*", "\\1", overdisp_output)

c(

colnames(Data)[i], # Pollutant name

summo$coefficients[2, 1], # Beta coefficient

summo$coefficients[2, 4], # p-value

summo$AICtab[1], # AIC

MI, # Moran's I

MI_p, # Moran's I p-value

ratio # Overdispersion ratio

)

}, warning = function(w) {

message(paste("Warning on iteration", i, ":", conditionMessage(w)))

return(NULL)

}, error = function(e) {

message(paste("Error on iteration", i, ":", conditionMessage(e)))

return(NULL)

})

}

MM_results <- as.data.frame(MM_loop)

colnames(MM_results) <- c(

"Variable",

"Beta",

"P_value",

"AIC",

"Morans_I",

"Morans_I_P",

"Overdispersion_Ratio"

)

MM_results[, 2:7] <- lapply(MM_results[, 2:7], as.numeric)

end_time <- Sys.time()

runtime <- difftime(end_time, start_time, units = "mins")

message(paste("Disease", ICD, "completed in", round(runtime, 2), "minutes"))

return(MM_results)

}

# Set up parallel processing

cl <- makeCluster(detectCores() - 1)

registerDoParallel(cl)

# Initialize output list

all_results <- list()

# Loop through all diseases

for (disease_code in names(disease_df)) {

message(paste("Processing disease:", disease_code))

results <- Mixed_Effect(disease_code)

all_results[[disease_code]] <- results

}

# Stop parallel cluster

stopCluster(cl)

# Combine all results

final_output <- bind_rows(all_results, .id = "Disease")

# Write to CSV

write.csv(final_output, "Spatial_Model_Results.csv", row.names = FALSE)

**REFERENCES**

1. Ratley G, Zeldin J, Sun AA, Yadav M, Chaudhary PP , Myles IA. Spatial modeling connecting childhood atopic dermatitis prevalence with household exposure to pollutants. Commun Med (Lond) 2024;4:74.

2. Zeldin J, Tran TT, Yadav M, Chaudhary PP, D'Souza BN, Ratley G et al. Antimony Compounds Associate with Atopic Dermatitis and Influence Models of Itch and Dysbiosis. Environ Sci Technol Lett 2023;10:452-7.

3. Zeldin J, Chaudhary PP, Spathies J, Yadav M, D'Souza BN, Alishahedani ME et al. Exposure to isocyanates predicts atopic dermatitis prevalence and disrupts therapeutic pathways in commensal bacteria. Sci Adv 2023;9:eade8898.

4. Team RC. R: A language and environment for statistical computing. R Foundation for Statistical Computing 2021.

**R Package References**

1. Analytics R, Weston S. iterators: Provides Iterator Construct. R package version 1.0.14, 2022. https://CRAN.R-project.org/package=iterators.
2. Attali D, Sali A. shinycssloaders: Add Loading Animations to a 'shiny' Output While It's Recalculating. R package version 1.1.0, 2024. https://CRAN.R-project.org/package=shinycssloaders.
3. Bartoń K. MuMIn: Multi-Model Inference. R package version 1.46.0, 2022. https://CRAN.R-project.org/package=MuMIn.
4. Bates D, Mächler M, Bolker B, Walker S. Fitting Linear Mixed-Effects Models Using lme4. J Stat Softw 2015;67(1):1-48.
5. Bates D, Maechler M, Jagan M. Matrix: Sparse and Dense Matrix Classes and Methods. R package version 1.6-5, 2024. https://CRAN.R-project.org/package=Matrix.
6. Bivand R, Nowosad J, Lovelace R. spData: Datasets for Spatial Analysis. R package version 2.3.4, 2025. https://CRAN.R-project.org/package=spData.
7. Bivand R, Wong D. Comparing implementations of global and local indicators of spatial association. TEST 2018;27(3):716-48.
8. Bivand R. R Packages for Analyzing Spatial Data: A Comparative Case Study with Areal Data. Geogr Anal 2022;54(3):488-518.
9. Bivand R, Pebesma E, Gómez-Rubio V. Applied spatial data analysis with R, Second edition. New York: Springer, 2013.
10. Pebesma E, Bivand R. Spatial Data Science With Applications in R. Chapman & Hall, 2023.
11. Chang W. shinythemes: Themes for Shiny. R package version 1.2.0, 2021. https://CRAN.R-project.org/package=shinythemes.
12. Chang W, Cheng J, Allaire J, Sievert C, Schloerke B, Xie Y et al. shiny: Web Application Framework for R. R package version 1.11.1, 2025. https://CRAN.R-project.org/package=shiny.
13. Corporation M, Weston S. doParallel: Foreach Parallel Adaptor for the 'parallel' Package. R package version 1.0.17, 2022. https://CRAN.R-project.org/package=doParallel.
14. Friedman J, Hastie T, Tibshirani R. Regularization Paths for Generalized Linear Models via Coordinate Descent. J Stat Softw 2010;33(1):1-22.
15. Simon N, Friedman J, Hastie T, Tibshirani R. Regularization Paths for Cox's Proportional Hazards Model via Coordinate Descent. J Stat Softw 2011;39(5):1-13.
16. Tay JK, Narasimhan B, Hastie T. Elastic Net Regularization Paths for All Generalized Linear Models. J Stat Softw 2023;106(1):1-31.
17. Grolemund G, Wickham H. Dates and Times Made Easy with lubridate. J Stat Softw 2011;40(3):1-25.
18. Hervé M. RVAideMemoire: Testing and Plotting Procedures for Biostatistics. R package version 0.9-83-12, 2025. https://CRAN.R-project.org/package=RVAideMemoire.
19. Hijmans R. raster: Geographic Data Analysis and Modeling. R package version 3.6-32, 2025. https://CRAN.R-project.org/package=raster.
20. Jackman S. pscl: Classes and Methods for R Developed in the Political Science Computational Laboratory. R package version 1.5.9, 2024. Sydney: University of Sydney. https://github.com/atahk/pscl/.
21. Zeileis A, Kleiber C, Jackman S. Regression Models for Count Data in R. J Stat Softw 2008;27(8).
22. Jawaid W. enrichR: Provides an R Interface to 'Enrichr'. R package version 3.4, 2025. https://CRAN.R-project.org/package=enrichR.
23. Kolde R. pheatmap: Pretty Heatmaps. R package version 1.0.13, 2025. https://CRAN.R-project.org/package=pheatmap.
24. Kuhn M. Building Predictive Models in R Using the caret Package. J Stat Softw 2008;28(5):1-26.
25. Makowski D, Lüdecke D, Patil I, Thériault R, Ben-Shachar M, Wiernik B. Automated Results Reporting as a Practical Tool to Improve Reproducibility and Methodological Best Practices Adoption. CRAN, 2023. https://easystats.github.io/report/.
26. Microsoft, Weston S. foreach: Provides Foreach Looping Construct. R package version 1.5.2, 2022. https://CRAN.R-project.org/package=foreach.
27. Müller K, Wickham H. tibble: Simple Data Frames. R package version 3.3.0, 2025. https://CRAN.R-project.org/package=tibble.
28. Pebesma E, Bivand R. Classes and methods for spatial data in R. R News 2005;5(2):9-13.
29. Pebesma E, Bivand R. Spatial Data Science: With applications in R. Chapman and Hall/CRC, 2023.
30. Pebesma E. Simple Features for R: Standardized Support for Spatial Vector Data. R J 2018;10(1):439-46.
31. Perrier V, Meyer F, Granjon D. shinyWidgets: Custom Inputs Widgets for Shiny. R package version 0.9.0, 2025. https://CRAN.R-project.org/package=shinyWidgets.
32. R Core Team. R: A Language and Environment for Statistical Computing. Vienna, Austria: R Foundation for Statistical Computing, 2024. https://www.R-project.org/.
33. Rozzi GC. zipcodeR: Advancing the analysis of spatial data at the ZIP code level in R. Softw Impacts 2021;9:100099.
34. Sarkar D. Lattice: Multivariate Data Visualization with R. New York: Springer, 2008.
35. Sievert C. Interactive Web-Based Data Visualization with R, plotly, and shiny. Chapman and Hall/CRC, 2020.
36. Wickham H. Reshaping Data with the reshape Package. J Stat Softw 2007;21(12):1-20.
37. Wickham H. ggplot2: Elegant Graphics for Data Analysis. New York: Springer-Verlag, 2016.
38. Wickham H. forcats: Tools for Working with Categorical Variables (Factors). R package version 1.0.0, 2023. https://CRAN.R-project.org/package=forcats.
39. Wickham H. stringr: Simple, Consistent Wrappers for Common String Operations. R package version 1.5.1, 2023. https://CRAN.R-project.org/package=stringr.
40. Wickham H, Averick M, Bryan J, Chang W, McGowan LD, François R et al. Welcome to the tidyverse. J Open Source Softw 2019;4(43):1686.
41. Wickham H, François R, Henry L, Müller K, Vaughan D. dplyr: A Grammar of Data Manipulation. R package version 1.1.4, 2023. https://CRAN.R-project.org/package=dplyr.
42. Wickham H, Henry L. purrr: Functional Programming Tools. R package version 1.1.0, 2025. https://CRAN.R-project.org/package=purrr.
43. Wickham H, Hester J, Bryan J. readr: Read Rectangular Text Data. R package version 2.1.5, 2024. https://CRAN.R-project.org/package=readr.
44. Wickham H, Vaughan D, Girlich M. tidyr: Tidy Messy Data. R package version 1.3.1, 2024. https://CRAN.R-project.org/package=tidyr.
45. Xie Y, Cheng J, Tan X. DT: A Wrapper of the JavaScript Library 'DataTables'. R package version 0.33, 2024. https://CRAN.R-project.org/package=DT.
